# The effects of metal artifact reduction on the retrieval of attenuation values

**DOI:** 10.1002/acm2.12002

**Published:** 2016-12-05

**Authors:** Christian Ziemann, Maik Stille, Florian Cremers, Dirk Rades, Thorsten M. Buzug

**Affiliations:** ^1^ University Hospital Schleswig Holstein Department of Radiotherapy / Campus Luebeck Department of Radiotherapy Ratzeburger Allee 160 D‐23562 Luebeck Germany; ^2^ University of Luebeck Institute of Medical Engineering Ratzeburger Allee 160 D‐23562 Luebeck Germany

**Keywords:** hounsfield units, implants, metal artifact reduction, prosthesis, radiotherapy

## Abstract

**Background:**

The quality of CT slices can be drastically reduced in the presence of high‐density objects such as metal implants within the patients’ body due to the occurrence of streaking artifacts. Consequently, a delineation of anatomical structures might not be possible, which strongly influences clinical examination.

**Purpose:**

The aim of the study is to clinically evaluate the retrieval of attenuation values and structures by the recently proposed Augmented Likelihood Image Reconstruction (ALIR) and linear interpolation in the presence of metal artifacts.

**Material and Methods:**

A commercially available phantom was equipped with two steel inserts. At a position between the metal rods, which shows severe streaking artifacts, different human tissue‐equivalent inserts are alternately mounted. Using a single‐source computer tomograph, raw data with and without metal rods are acquired for each insert. Images are reconstructed using the ALIR algorithm and a filtered back projection with and without linear interpolation. Mean and standard deviation are compared for a region of interest in the ALIR reconstructions, linear interpolation results, uncorrected images with metal rods, and the images without metal rods, which are used as a reference. Furthermore, the reconstructed shape of the inserts is analyzed by comparing different profiles of the image.

**Results:**

The measured mean and standard deviation values show that for all tissue classes, the metal artifacts could be reduced using the ALIR algorithm and the linear interpolation. Furthermore, the HU values for the different classes could be retrieved with errors below the standard deviation in the reference image. An evaluation of the shape of the inserts shows that the reconstructed object fits the shape of the insert accurately after metal artifact correction. Moreover, the evaluation shows a drop in the standard deviation for the ALIR reconstructed images compared to the reference images while reducing artifacts and keeping the shape of the inserts, which indicates a noise reduction ability of the ALIR algorithm.

**Conclusion:**

HU values, which are distorted by metal artifacts, can be retrieved accurately with the ALIR algorithm and the linear interpolation approach. After metal artifact correction, structures, which are not perceptible in the original images due to streaking artifacts, are reconstructed correctly within the image using the ALIR algorithm. Furthermore, the ALIR produced images with a reduced noise level compared to reference images and artifact images. Linear interpolation results in a distortion of the investigated shapes and features remaining streaking artifacts.

## 1 Introduction

Computed tomography (CT) continues to be one of the key methods in medical imaging.^1^, ^2^ This is especially the case for radiation therapy, where a CT scan is the basis of radiation treatment planning. Within this process, Hounsfield units (HU) are translated into electron densities, which are essential to calculate dose distributions. Furthermore, qualitatively and quantitatively sufficient imaging is required for the differentiation and segmentation of regions being treated and organs at risk, which should be spared. Unfortunately, the image quality of reconstructed CT slices can be reduced by the occurrence of different artifacts.^3^ One of the main sources for artifacts is the presence of objects with a high density, that is, prostheses, dental implants, or surgical tools.^3^, ^4^ Due to various physical effects such as scatter, beam hardening, noise, or total absorption, projections that pass through such an object can become useless for the reconstruction of the scanned object. This leads to incorrectly reproduced HU values, which in turn, affect the dose calculation.^5^, ^6^ Image quality is potentially being reduced up to a point where a delineation of anatomical structures is no longer possible. This drastically influences the clinical examination.^5^, ^7^, ^8^, ^9^ Consequently, an accurate contouring of target structures and organs at risk is no longer guaranteed and the dose planning process is inaccurate.

The correction of metal artifacts remains a highly active field with many different approaches being published every year.^10^ However, since publication of the linear interpolation (LI) approach in 1987, only a few advanced methods with a high clinical potential have been proposed.^8^, ^11^, ^12^, ^13^, ^14^ One particular method of interest is the Augmented Likelihood Image Reconstruction (ALIR) that has proven to outperform current methods for clinically relevant data.^15^ In order to integrate such a method in the daily routine within a clinical environment, the method needs to be evaluated extensively.^5^, ^7^, ^16^, ^17^ Such evaluation should not only focus on retrieving missing anatomical information and improving image quality, but should also investigate the retrieval of correct HU values. Studies that evaluate the performance of MAR methods such as iMAR or VME used tissue‐equivalent inserts in phantoms in order to study the HU value retrieval.^7^, ^18^ Comparisons of the MAR methods with undisturbed reference images showed that the original HU values could be approximated. In most of the cases, the noise was reduced, while in other cases, the noise was also partly increased.^7^, ^18^ Evaluation of the MAR algorithms with respect to their correction capabilities of HU values was slightly limited due to the fact that examined inserts were not alternately positioned on the position with the highest amount of distortion. Therefore, the degree of artifact severity differs for each insert. For a meaningful evaluation, the amount of artifacts should be approximately the same for each insert, which can only be achieved if each insert is positioned at the same location with respect to the metal objects.

Since the ALIR algorithm has already been applied to patient data and has proven that anatomical details can be reconstructed accurately within a complex evaluation in cooperation with radiologists, the algorithm is intensively studied with a focus on the correct retrieval of attenuation values. Here, a commercially available phantom, which is utilized for clinical calibration, is used in order to evaluate the performance of the ALIR algorithm and the LI approach. Different tissue‐equivalent inserts are mounted between two metal rods and the reconstructed HU values are analyzed before and after metal artifact reduction. All values are compared to reference images that are acquired without metal rods. Furthermore, the retrieval of the shape of the inserts is analyzed based on the profile plots and a comparison with reference images. Since the present study is limited to phantom data, the reader is referenced to the expensive evaluation of the ALIR algorithm on clinical data in.^15^

## 2 Material and methods

### 2.A Phantom

A commercially available heterogeneous phantom (Gammex Electron Density CT Phantom Model 465, Radiation Measurements Inc.; Middleton, USA) was used in order to represent different human tissue classes. The main body of the phantom is composed of Solid Water and is equipped with 20 holes with a diameter of 30 mm each. The holes can be used to mount different tissue‐equivalent cylindrical rods with known mass density and an electron density relative to water, denoted by $\rho_{e}^{W}$. A wide range of tissue‐equivalent inserts, from cortical bone with $\rho_{e}^{W}=1.707$ to lung 300 with $\rho_{e}^{W}=0.292$ was used in order to investigate the reconstruction ability of the Augmented Likelihood Image Reconstruction algorithm and the linear interpolation approach.^11^, ^15^

**Figure 1:**
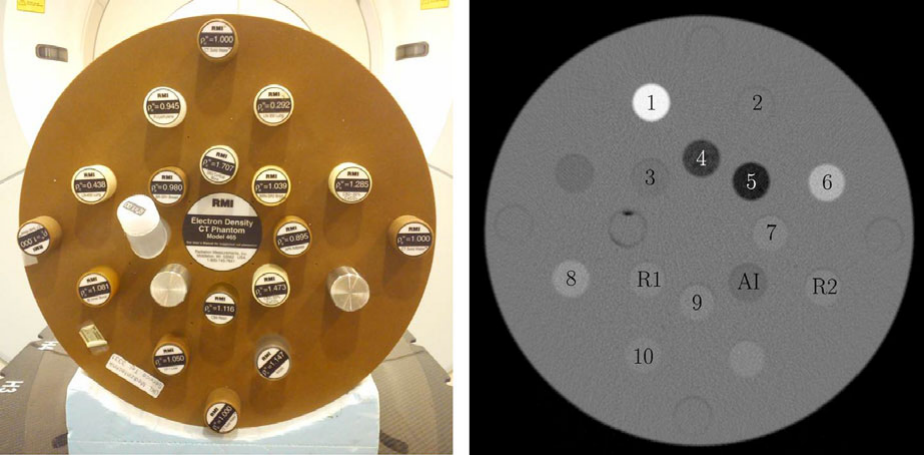
A photo of the used phantom with metal inserts, shown on the left side. On the right side, a labeled CT image with the tissue‐equivalent electron densities: (1) cortical bone, (2) brain tissue, (3) breast tissue, (4) lung 450, (5) lung 300, (6) CaCO3, (7) adipose, (8) inner bone, (9) bone mineral, (10) liver tissue, (*R1*) and (*R2*), which show the position of the metal rods, and (*AI*) which shows the position of interest.

Figure 1 shows the used phantom and a CT image with labeled positions. In order to simulate a double‐sided hip prosthesis, which causes severe artifacts, metal rods were manufactured and mounted at position *R1* and *R2*. At the position between the metal rods, where the strongest manifestation of the artifacts is presumed, the tissue‐equivalent inserts where alternately inserted (position is labeled *AI*). The original position of the insert that currently occupies position *AI*, is filled with an insert that has a density close to Solid Water. This setup results in ten different configurations where each of the ten tissue‐equivalent inserts occupies position *AI*. A CT scan with and without mounted metal rods is performed for each configuration. The resulting holes at *R1* and *R2* are again filled with Solid Water inserts for the metal‐free acquisition. The metal‐free images are used as ground truth for the evaluation of the metal artifact reduction and are further denoted as the reference images.

### 2.B CT imaging

A 40‐slice Biograph mCT (Siemens AG; Erlangen, Germany) was used for the acquisition of the images. The disk‐shaped phantom was positioned at the isocenter using a pedestal consisting of Styrodur^**®**^, which secured the phantom against changes in position during table motion and replacement of the inserts.

Scans were acquired sequentially with a slice thickness of 3 mm, a voltage of 120 kV, a field of view of 500 mm, and the flying focal spot setting enabled.

### 2.C Reduction of artifacts

For the reduction of metal artifacts, LI and the recently proposed ALIR algorithm is used.^11^, ^15^ The ALIR algorithm is based on an iterative scheme and integrates two different ideas in order to reduce streaking artifacts. The reconstruction of an image is modeled as an optimization problem, which utilizes the negative log‐likelihood function for transmission CT as the objective.^3^ In addition to the objective, the algorithm integrates constraints that force the reconstruction to assign certain attenuation values in the region of the metal implant. These could be either known attenuation values of the metal implant, which could be gained by utilizing a computer‐aided design (CAD) description of the implant, or arbitrary values defined by the user. In the present case, the attenuation value for water is used for the location of the metal object.

The second approach for the reduction of streaking artifacts, which is integrated in ALIR, is based on the interim results of the reconstruction. Let *f* ^(*k*)^ be the image that can be obtained in the *k*th iteration. Temporarily appearing artifacts are reduced by applying a bilateral filter to the image *f* ^(*k*)^.^19^ The filter has two parameters, a geometric spread, *σ*_*d*_, and a photometric spread, *σ*_*r*_, which can both be adjusted to the manifestation of the artifacts. However, both parameters are fixed for all performed reconstructions. Based on the filtered image *g* ^(*k*)^ in iteration *k*, it is now possible to calculate new projection values. These projection values are used in iteration *k + 1* instead of the acquired values, which are corrupted due to the high density of the metal object. Over the total reconstruction, the newly calculated projection values contain more and more information of the anatomy of the patient in every iteration. In this way, sufficiently filled raw data can be obtained and an image with reduced artifacts can be reconstructed. The algorithm stops when the gradient of the objective reaches a sufficiently small value. The algorithm reached convergence in less than 20 iterations for all results shown. A more detailed explanation of the ALIR algorithm together with an extensive evaluation on clinical image data is given in.^15^

### 2.D Evaluation methods

#### 2.D.1 Analysis of hounsfield unit retrieval

A rectangular region of interest (ROI) with a size of 16 × 16 pixels, which corresponds to an area of 16.38 × 16.38 mm^2^, was defined and positioned into the center of position AI. The coordinates of the ROI were transferred to all available data sets, that is, reference, uncorrected, LI corrected images, and ALIR corrected images of each exchanged tissue‐equivalent insert at position AI. For each image, the statistical parameters mean and standard deviation of the HU values are calculated for the ROI.

#### 2.D.2 Profile analysis

In order to analyze the behavior of the ALIR and LI algorithm regarding the smoothness of the resulting image and the preservation of the edge sharpness, a profile of a ROI within the images is examined. Due to the mounting procedure of the metal objects between the acquisitions of the images, small movements of the phantom can occur. To ensure an accurate overlap of the different image types, that is, metal artifact corrected images, image with artifacts, and reference, an affine registration is performed using the Insight Segmentation and Registration Toolkit (ITK).^20^, ^21^ With the area of the metal object masked out, a cost function based on correlation is used. After alignment, a region of interest was chosen that shows changes in the attenuation values and a profile was extracted (see Figs. 3 and 4). In order to analyze the sharpness of edges, the derivative of the profile function was calculated to give information about the slope in these areas.

## 3 Results

**Figure 2:**
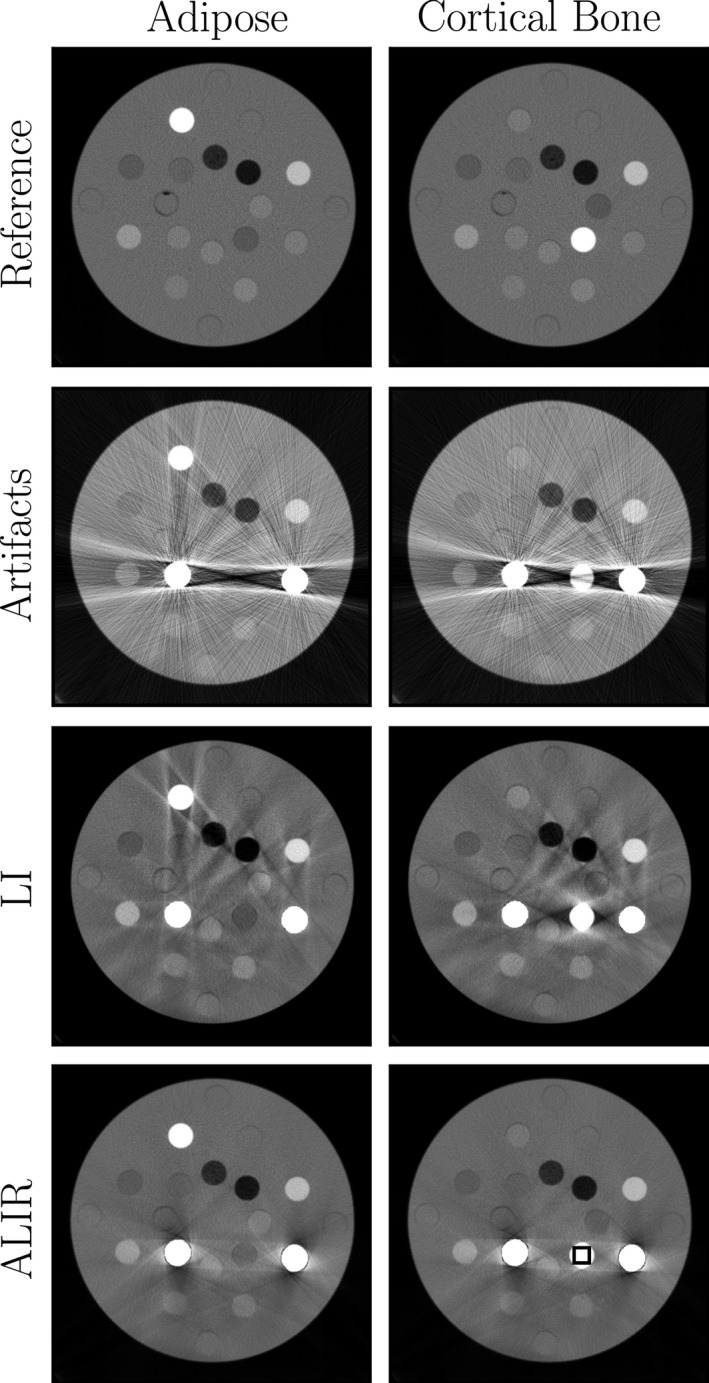
Two example image sets for the tissue‐equivalent inserts for adipose and cortical bone. First row shows the reference images without metal rods. Second row shows the uncorrected images, where metal rods were mounted on position *R1* and *R2*. Third row shows the linear interpolation (LI) results. The last row shows images that are reconstructed with the ALIR algorithm in order correct metal artifacts. Images are shown with a window level of 1600 HU and a window width of 1400 HU. The black rectangle shows the ROI that is used for evaluation of the attenuation values.

In Fig. 2, two example image sets of the used phantom are shown. In the first column, the tissue‐equivalent insert for adipose is mounted at position *AI* and on position 7, a Solid Water insert is placed. In the first row, the reference image is shown, where the metal rods are replaced by Solid Water inserts. This reference image gives information about the exact HU values that should be reconstructed at this position. In the second row, images are shown, where metal rods are mounted at position *R1* and *R2*. These images show strong streaking artifacts over the entire image. Especially, the very pronounced beam‐hardening artifact that connects the two metal rods prevents a differentiation of the different tissue‐equivalent inserts. It is almost not discernible that position *AI* shows a different structure than its surrounding. The third row shows the result from the linear interpolation (LI) approach. Although the strong beam hardening artifact could be reduced, a lot of streaking artifacts are remaining. The shape of the insert at position *AI* can be well spotted. However, the shape of the insert is rather oval than circular. Only after metal artifact correction with the ALIR algorithm, the adipose equivalent insert is clearly perceptible as are all other tissue‐equivalent inserts.

A very similar situation can be observed in the case of the cortical bone equivalent insert at position *AI*. Again a Solid Water insert is mounted at the original position of the insert at position 1. Due to the high $\rho_{e}^{W}$ value for the insert, the object is vaguely perceptible at position *AI* in the image with the metal rods. The LI result shows severe artifacts around position *AI* and many remaining streaking artifacts. Only after metal artifact reduction with the ALIR algorithm, the correct shape and HU values can be observed. However, some artifacts around the metal objects are remaining.

**Figure 3:**
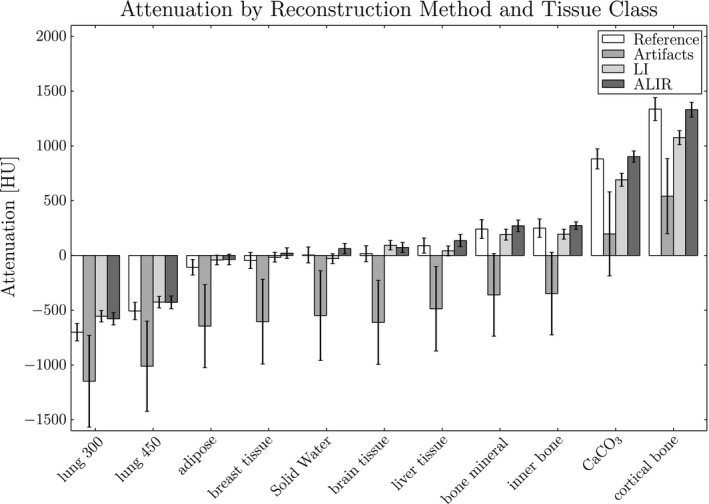
Attenuation coefficients for the different reconstruction methods and the used tissue classes. Black lines indicate the standard deviation of the measured values.

Table 1 shows the mean values, *μ*, and the standard deviation, *σ*, of the analyzed ROI for the reference images, the uncorrected images, the LI results and the ALIR reconstructed images. Furthermore, the values are visualized within a boxplot in Fig. 3. The used ROI is shown in black in Fig. 2. As expected, due to the pronounced streaking artifacts, the mean values for all tissue classes in case of the uncorrected images are far off compared to the reference values. After LI, the mean values come closer to the reference values with a minimum error of 30.10 HU for breast tissue and a maximum error of 260.40 HU for cortical bone.

**Table 1:** Mean values, *μ*, for all tissue‐equivalent inserts at position *AI* for the reference, metal artifact corrected and not corrected images. Furthermore, the standard deviation, *σ*, is shown for all images

Tissue class	$\rho_{e}^{W}$	*μ*_ref_ [HU]	*μ*_metal_ [HU]	*μ*_LI_ [HU]	*μ*_ALIR_ [HU]	*σ*_ref_ [HU]	*σ*_metal_ [HU]	*σ*_LI_ [HU]	*σ*_ALIR_ [HU]
Lung 300	0.292	−700.6	−1148.0	−554.8	−578.1	79.4	418.8	52.2	55.6
Lung 450	0.450	−507.1	−1011.0	−426.1	−428.2	79.0	411.8	52.4	58.5
Adipose	0.895	−107.7	−645.2	−41.8	−36.6	70.7	379.2	44.3	48.4
Breast tissue	0.980	−46.2	−604.4	−16.0	21.1	73.5	386.8	44.1	48.4
Solid water	1.000	4.5	−548.6	−28.9	62.6	72.6	409.4	44.6	47.2
Brain tissue	1.039	16.2	−611.1	93.3	73.0	72.8	383.5	43.3	45.6
Liver tissue	1.050	89.5	−487.1	42.1	135.8	69.2	385.1	45.6	55.2
Bone mineral	1.070	240.7	−359.9	190.1	270.7	86.4	376.9	49.8	53.1
Inner bone	1.081	249.8	−348.2	194.5	273.1	84.0	376.8	45.5	33.6
CaCO_3_	1.285	881.3	197.1	690.5	902.2	91.6	383.6	59.2	51.3
Cortical bone	1.707	1336.0	541.5	1075.6	1331.0	105.0	342.4	64.4	67.8

Reconstructed image resulting from the ALIR algorithm show mean values that come very close to the reference values. Especially for inserts with a high $\rho_{e}^{W}$ value, the attenuation values could be reconstructed in an accurate manner. The resulting error is mostly smaller than the standard deviation for the corresponding tissue class with a minimum error of 5.00 HU for cortical bone and a maximum error of 122.50 HU for lung 300.

**Figure 4:**
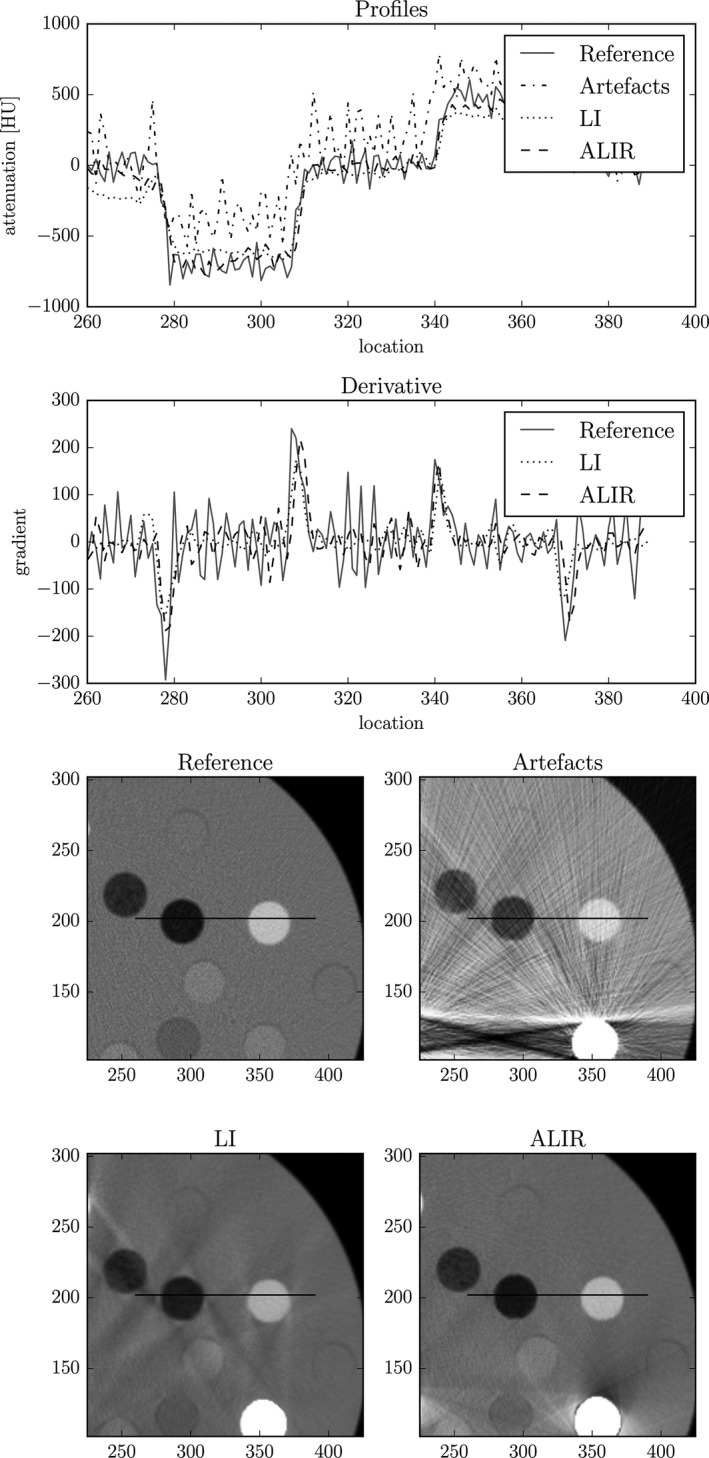
Profile plot of a region of interest for the metal artifact corrected images, the reference image and the image that shows artifacts. Additionally, the derivative of the profile function is shown in order to represent the sharpness of the edges. Corresponding images are shown in a window width of 1150 HU with a level of 1350 HU.

**Figure 5:**
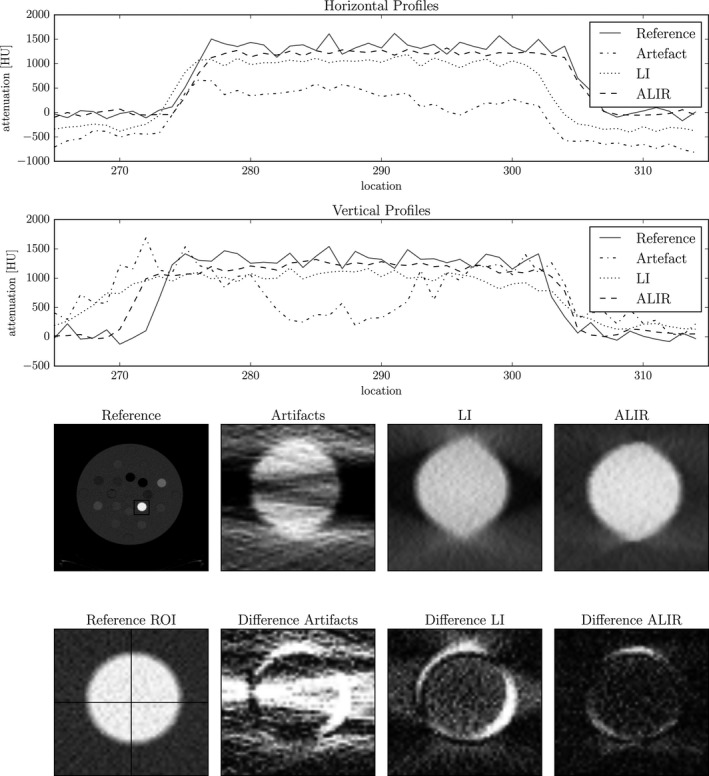
Vertical and horizontal profile plot for the cortical bone equivalent insert. Bottom row shows the corresponding ROI of the different reconstructions. Differences are relative to the reference image. Reference and corrected images are shown in a window width of 1000 HU at window level 1500 HU. The image with artifacts is shown with a window width of 2000 HU at a level of 2000 HU. The difference images are shown with a window width of 1000 HU at level 1000 HU.

Furthermore, while the standard deviation for the images with metal artifacts are very high due to the amplified noise and pronounced streaking artifacts, the standard deviation for all tissue classes after metal artifact reduction is smaller than the reference values. This indicates that not only a reduction in streaking artifacts could be gained but also a reduction in the noise level is achieved while the mean values of the individual tissue class is preserved. However, for the LI images, this result comes with a drawback. Images show a much smoother overall appearance, that is, not only the standard deviation is reduced in homogeneous areas but the edges in the images are also smoothed as it can be seen in Figs. 4 and 5. On the other hand, the edges in the ALIR results preserve a sharp appearance.

In order to investigate whether the shape of the inserts remains unchanged compared to the reference image, a profile plot of the inserts is examined. Fig. 4 shows the profile of a ROI that features lung 300 and CaCO_3_ equivalent tissue at position 5 and 6, respectively. The profile for the image with artifacts features an offset of approx. 300 HU, which is caused by the pronounced streaking artifacts. Furthermore, the perception of edges between phantom body and inserts is heavily affected by streaking artifacts and amplified noise (see standard deviation in Table 1). The profile for LI features a smooth transition from the body of the phantom to the specific inserts. A streaking artifact can be seen between location 260 and 180 where the attenuation values are way below the reference values. Most importantly, the investigated region of interest features remaining streaking artifacts and shows a smooth appearance overall. However, images resulting from the ALIR algorithm show the same trend of the profile as the reference image. Furthermore, the noise level could be reduced, which is clearly obtainable in the smooth trend of the profile. Another indication for this behavior can be obtained in Table 1, which shows reduced standard deviation values for all tissue classes in the metal artifact corrected images compared to the reference images. In order to analyze the slope of the edges, the derivative of the profiles is generated. It can be obtained, that at the position of the edges within the profile, the derivative of the corrected and reference profile show similar amplitude. This clearly indicates that the edges are preserved in the images that are reconstructed with the ALIR algorithm. Furthermore, the amplitude of the derivative for LI show smaller values compared to the reference and ALIR results, which confirms the smooth appearance of the LI results around the edges. Since the profile for the artifact image shows such a high level of noise combined with the streaking artifacts, the derivative for this function is omitted.

With a focus on position AI, Fig. 5 shows profiles for the cortical bone‐equivalent insert. A vertical and horizontal profile is shown in order to analyze the shape of the insert. Due to the metal artifacts, the profile for the uncorrected image shows an almost arbitrary curve. Especially, the vertical profile gives no indication of a circular shaped insert. The calculated difference between the uncorrected image and the reference shows pronounced streaks and a high level of noise. After LI, noise is significantly reduced. However, the shape of the insert is not reconstructed correctly and shows an oval appearance. Remaining dark streaks can clearly be seen in the profiles. The vertical profiles show the very smooth transition from insert to the body of the phantom where no clear edge is recognizable. The calculated difference between reference and LI shows a high error along the whole edge of the insert. Remaining streaks are clearly recognizable. However, the ALIR algorithm is able to restore the shape of the insert accurately. The horizontal line shows a trend of the profile that fits the reference image almost exactly. The vertical profile reveals some remaining artifacts on the edge of the insert, which are caused by the pronounced streaking artifacts. This can also be seen in the calculated difference between the ALIR reconstruction and the reference image. Nonetheless, the calculated difference shows that a reasonable retrieval of the tissue‐equivalent insert could be achieved.

## 4 Discussion

### 4.A Retrieval of HU values

The correct retrieval of the HU values is possible due to the fact that the ALIR reconstruction uses mostly projection values, which are not influenced by metal. In order to reconstruct an image without the usage of x rays that run through a metal object, the ALIR reconstruction divides the set of projections indices ***M*** = {1, …, *M*} into a set of indices for projections that are not affected by metal, ***M***_**1**_, and a set for projections that are affected by metal, ***M***_**2**_, such that ***M*** = ***M***_**1**_ ∪ ***M***_**2**_ and $M_{1}\cap M_{2}=\phi$.^15^ More often than not the metal object is much smaller compared to patients anatomy shown in the image, therefore | ***M***_**2**_ | < | ***M***_**1**_ | .

Using a traditional reconstruction like the filtered back projection without any ability to reduce metal artifacts, the set ***M***_**2**_ is used as well as the set ***M***_**1**_. This causes artifacts, which superimpose the anatomical information and therefore the correct HU values. Iteratively, the set ***M***_**2**_ is replaced by filtered forward projections within ALIR. The newly calculated projection values are becoming more accurate in every iteration since more and more anatomical details are reconstructed and included within these projection values. Due to the bilateral filter, artifacts can be reduced and the resulting image contains less streaks. Consequently, the original attenuation values of the anatomical structures emerge.

Results for LI show a reasonable retrieval of HU values with a slightly higher error compared to the ALIR results. However, the overall reduction of streaks is inferior and comes with the drawback of smooth images, which can be seen especially at the edges of the inserts.

### 4.B Restoration of the shape of the inserts

Specifically for position AI, the set of projection values being affected by metal is relatively large. For ALIR, this has the effect that the reconstruction of the correct HU values in this area is highly influenced by the newly calculated projection values. Further, the outcome of the filtering procedure is highly influenced by the geometric and photometric spread. Artifacts which feature a strong edge compared to the anatomical information around it may not be smoothed or suppressed. In this case, the two parameters need to be adapted to the manifestation of the streaking artifacts. However, a high photometric and geometric spread might lead to a smoothing of edges that correspond to anatomical shapes, as well. Consequently, this results in a trade‐off between artifact reduction and preservation of the edges within the image. In a clinical setting, it is advisable to give the relevant clinician the option to change these parameters in order to produce the preferred outcome. For an evaluation of the ALIR algorithm on clinical data, the reader is kindly referenced to.^15^

### 4.C Reduction of noise

The reduced standard deviations after metal artifacts reduction, which are shown in Table 1, indicate a noise reduction in the images. For ALIR, this behavior can again be explained by means of the bilateral filter used in the algorithm. Controlled by the geometric and photometric spread, a smoothing step is specified for image *f* ^(*k*)^ in iteration *k*. The filter does not only reduce streaking artifacts but also smooths homogeneous areas where the attenuation values are within the photometric spread. Therefore, noise is reduced in the filtered image *g* ^(*k*)^ and transported further in the newly calculated projection values that are used in the reconstruction for iteration *k* + 1.

The LI approach also results in a reduction of noise but with the important drawback of an overall smoothing of the image, which is generally not desirable. Compared to ALIR, edges of inserts are smoothed and show occasionally a very gradual transition as can be seen in Fig. 4.

## 5 Conclusion

An evaluation of HU values that are distorted by metal artifacts is presented and investigated. The results show that it is possible to retrieve these values accurately with the ALIR algorithm. After metal artifact correction with ALIR, structures, which are not perceptible in the original images due to streaking artifacts, are reconstructed correctly within the image. Furthermore, ALIR results in images with a reduced standard deviation compared to the reference and artifact images. This indicates a promising noise reduction ability of the recently proposed algorithm and will be researched intensively in the near future.

The LI approach on the other hand results in a reasonable retrieval of HU values. However, images show an overall smooth appearance of structures, while the reduction of streaking artifacts is inferior compared to the ALIR algorithm.
